# Preoperative stereotactic radiosurgery in the management of brain metastases and gliomas

**DOI:** 10.3389/fsurg.2022.972727

**Published:** 2022-10-24

**Authors:** Eric J. Lehrer, Roman O. Kowalchuk, Henry Ruiz-Garcia, Kenneth W. Merrell, Paul D. Brown, Joshua D. Palmer, Stuart H. Burri, Jason P. Sheehan, Alfredo Quninoes-Hinojosa, Daniel M. Trifiletti

**Affiliations:** ^1^ Department of Radiation Oncology, Icahn School of Medicine at Mount Sinai, New York, NY, United States; ^2^ Department of Radiation Oncology, Mayo Clinic, Rochester, MN, United States; ^3^ Department of Radiation Oncology, Mayo Clinic, Jacksonville, FL, United States; ^4^ Department of Radiation Oncology, Ohio State University Wexner Medical Center, Columbus, OH, United States; ^5^ Department of Radiation Oncology, Atrium Health, Charlotte, NC, United States; ^6^ Department of Neurological Surgery, University of Virginia, Charlottesville, VA, United States; ^7^ Department of Neurosurgery, Mayo Clinic, Jacksonville, FL, United States

**Keywords:** brain metastases, glioma, stereotactic radiosurgery, neurosurgery, radiation oncology

## Abstract

Stereotactic radiosurgery (SRS) is the delivery of a high dose ionizing radiation in a highly conformal manner, which allows for significant sparing of nearby healthy tissues. It is typically delivered in 1–5 sessions and has demonstrated safety and efficacy across multiple intracranial neoplasms and functional disorders. In the setting of brain metastases, postoperative and definitive SRS has demonstrated favorable rates of tumor control and improved cognitive preservation compared to conventional whole brain radiation therapy. However, the risk of local failure and treatment-related complications (e.g. radiation necrosis) markedly increases with larger postoperative treatment volumes. Additionally, the risk of leptomeningeal disease is significantly higher in patients treated with postoperative SRS. In the setting of high grade glioma, preclinical reports have suggested that preoperative SRS may enhance anti-tumor immunity as compared to postoperative radiotherapy. In addition to potentially permitting smaller target volumes, tissue analysis may permit characterization of DNA repair pathways and tumor microenvironment changes in response to SRS, which may be used to further tailor therapy and identify novel therapeutic targets. Building on the work from preoperative SRS for brain metastases and preclinical work for high grade gliomas, further exploration of this treatment paradigm in the latter is warranted. Presently, there are prospective early phase clinical trials underway investigating the role of preoperative SRS in the management of high grade gliomas. In the forthcoming sections, we review the biologic rationale for preoperative SRS, as well as pertinent preclinical and clinical data, including ongoing and planned prospective clinical trials.

## Introduction

Stereotactic radiosurgery (SRS) was first proposed by Dr. Lars Leksell in 1951 (1). This technique delivers high doses of ionizing radiation in 1–5 sessions in a highly conformal manner that allows for significant sparing of nearby healthy tissues due to the rapid dose gradient outside of the treatment target (1–4). Today, SRS is utilized in a multitude of benign and malignant intracranial indications (5–35).

One of the most common indications for SRS is in the management of brain metastases (13, 16, 19, 36). Multiple randomized trials have demonstrated that SRS is associated with excellent rates of local tumor control and improved rates of cognitive preservation without compromising overall survival (OS) when compared to whole brain radiation therapy (WBRT) (13–15). Historically, patients with a single accessible brain metastasis often underwent surgical resection, which demonstrated improved OS when compared to WBRT alone (37). Additionally, postoperative WBRT has demonstrated improved local and distant brain control, as well as lower rates of neurologic death when compared to surgery alone (38). However, given the cognitive sequelae associated with WBRT, clinicians frequently withheld it in the up-front setting (39). A recent randomized controlled trial comparing adjuvant SRS to observation in the setting of a resected brain metastasis demonstrated a 1 year local control rate of 72% in the SRS arm vs. 43% in the observation arm; however, the findings in the SRS arm were largely dependent on the size of the metastasis, as larger lesions were associated with worse local control (16).

Postoperative SRS is associated with several drawbacks, despite the improvement in local control. First, the use of a clinical target volume (CTV) expansion of 1 mm to 2 mm is commonly utilized to address microscopic, invasive disease. Second, postoperative SRS frequently requires that target volumes that encompass the surgical tract, as well as margin along the bone flap and venous sinuses (40). Taken together, these factors result in an increase in irradiated volume of normal brain, which is associated with an increased risk of treatment-related complications (e.g., radionecrosis) (8, 41–44). Third, the risk of leptomeningeal disease (LMD) is higher in patients undergoing postoperative SRS, likely due to surgical perturbation, compared to WBRT with rates as high as 45% (16, 45–49). Fourth, adherence rates with postoperative SRS are often suboptimal due to variable postoperative clinical courses (49, 50). Fifth, prolonged intervals between surgical resection and postoperative SRS are associated with worse local control (51–53).

Historically, radiation was given following surgery for resected brain metastases, but with the associated drawbacks of postoperative SRS, investigators began to explore incorporation of SRS in the preoperative setting. Preoperative therapy has become widely adopted in multiple malignancies, such as cancers of the esophagus and rectum (54–57). While most studies exploring the role of preoperative radiosurgery have focused on brain metastases, there has been recent growing interest in applying this treatment paradigm to high grade glioma and glioblastoma (11). While the use of postoperative SRS in the management of glioblastoma yielded disappointing results (58–60); however, its use in the preoperative setting shares many of the potential advantages observed with brain metastases and further might also be used as a strategy to enhance anti-tumor immunity (61, 62). Furthermore, preoperative SRS allows for post-radiotherapy tissue analysis, which can allow for characterization of DNA repair pathways and tumor microenvironment changes in response to SRS.

## Brain metastases

Brain metastases are the most common intracranial neoplasm and are diagnosed in approximately 200,000 patients each year in the United States (63–66). These estimates are likely conservative, as brain metastases are commonly diagnosed during the disease course, while national registries (e.g., The National Cancer Database and Surveillance, Epidemiology, and End Results) are largely focused on clinical characteristics present at the time of index cancer diagnosis (67, 68). For many years the standard of care treatment approach in these patients consisted of conventional whole-brain radiation therapy (WBRT) with or without resection and corticosteroids (69). In the absence of surgical resection many patients did not live beyond 3–4 months (37). With advances in systemic therapy (e.g., immune checkpoint inhibitors) the prognosis of patients with brain metastases has markedly improved (5–7, 70–72). Furthermore, greater availability of magnetic resonance imaging has increased detection of subclinical disease. Taken together, the incidence of brain metastases is expected increase, as well as the need for improved intracranial RT delivery.

### Surgical management of brain metastases

In 1990, Patchell et al. published a landmark randomized trial, where 48 patients with a single brain metastasis were randomized to surgical resection followed by postoperative WBRT or needle biopsy followed by WBRT (37). Whole brain radiation therapy was delivered to a dose of 36 Gy in 12 fractions. Patients who received surgical resection experienced improved lower rates of local recurrence (20% vs. 52%; *p* < 0.02), as well as improved median OS (40 weeks vs. 15 weeks; *p* < 0.01), and longer period of functional independence (median, 38 weeks vs. 8 weeks; *p* < 0.005).

### Postoperative radiotherapy for brain metastases

In 1998, Patchell et al. published the results of a randomized study designed to determine if WBRT improved neurologic outcomes and OS (38). Ninety-five patients with a single brain metastasis who underwent surgical resection were randomized to WBRT to a dose of 50.4 Gy in 28 fractions or observation. Patients in the WBRT arm experienced improved brain control (18% vs. 70%; *p* < 0.001), lower rates of local recurrence (10% vs. 46%; *p* < 0.001), and lower rates of neurologic death (14% vs. 44%; *p* = 0.003). No differences were observed in OS and length of functional independence. The results of the two Patchell studies established the role of postoperative WBRT in the management of a resected brain metastasis.

While WBRT is associated with excellent rates of local and regional brain control, it is also associated with significant rates of cognitive deterioration following treatment (13–15, 73–76). Multiple studies have suggested that there is an association between cognitive functioning and quality of life (77, 78). In 2017, Brown et al. published the results of N107C, which was a phase 3 trial that randomized 194 patients to SRS or WBRT following surgical resection of a brain metastasis (13). Overall cognitive deterioration was 52% vs. 85% (*p* = 0.00031) favoring the SRS arm and 12 month surgical bed control favored the WBRT arm (60.5% vs. 80.6%; *p* = 0.00068). In 2018, Mahajan et al. published the results of a single institution trial that randomized patients following resection of a brain metastasis to postoperative SRS or observation (16). At 12 months, the local control rates were 43% vs. 72% (*p* = 0.015), favoring the SRS arm with no differences in OS observed between the arms. Additionally, the local control rates were highly dependent on tumor diameter. When compared to a tumor diameter of ≤ 2.5 cm, tumors measuring > 2.5–3.5 cm [hazard ratio (HR): 6.7; *p* = 0.0021] and > 3.5 cm (HR: 6.6; *p* = 0.0032) had a markedly higher rate of local recurrence. Thus, when managing larger lesions, fractionated radiosurgery is a commonly utilized approach (8, 42, 43) and is being studied in an ongoing prospective randomized trial (79). Taken together, these studies have established the role of postoperative SRS in the setting of a resected brain metastasis.

**Figure 1:**
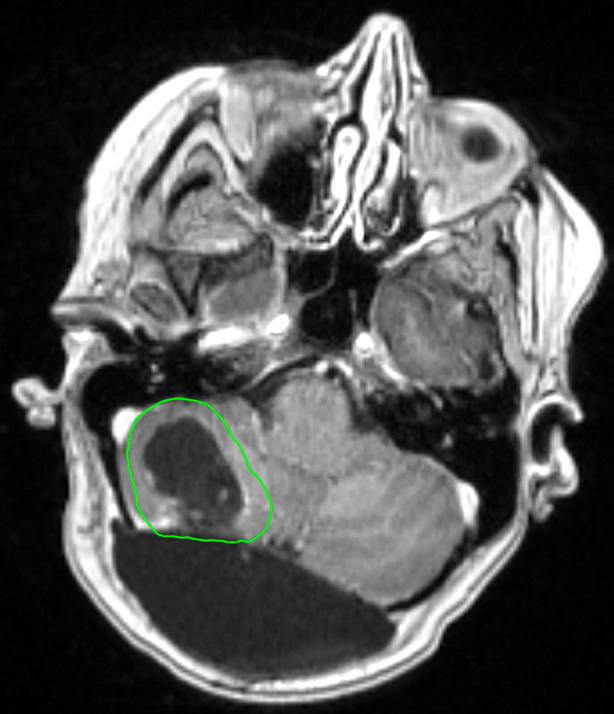
Radiation treatment plan of a 65 year old female patient with a history of breast cancer. She underwent a surgical resection followed by postoperative SRS to the resection cavity (outlined in green) to a dose of 27 Gy in 3 fractions.

The development of LMD is a significant concern in patients with brain metastases, particularly following surgical resection. Mahajan et al. observed an approximately 25% LMD rate in the postoperative SRS arm (16). In a 2017 study by Foreman et al., a 35% LMD rate at 1 year following postoperative SRS was observed (47). Additionally, they observed trend towards an increased risk of developing LMD in patients with breast cancer histology (HR: 2.37; *p* = 0.07). A study by Atalar et al. that evaluated 175 brain metastasis resection cavities that were treated with postoperative SRS observed an 11% cumulative incidence of LMD at 1 year (46). They also noted a 24% LMD rate in breast cancer patients compared to 9% in patients with other histologies (*p* = 0.004). Furthermore, resection and postoperative SRS is associated with a particular subtype of LMD, known as nodular LMD (nLMD) (45, 80). A radiation treatment plan for a patient who underwent resection and postoperative SRS is presented in Figure 1.

### Preoperative radiosurgery for brain metastases

**Figure 2:**
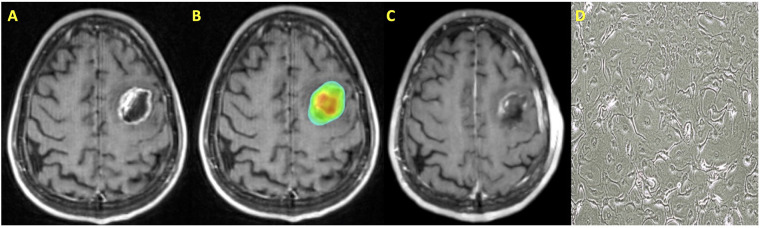
Patient with a history of metastatic non-small cell lung cancer who was treated with preoperative SRS. (**A**) T1 post contrast MRI axial image demonstrating a left frontal metastasis; (**B**) SRS treatment plan with dose color wash; (**C**) T1 post contrast MRI axial image following surgical resection; (**D**) Cell culture microscopy of post-irradiation resected tissue.

Preoperative SRS is a treatment strategy that may mitigate the risk of treatment-related toxicities and local failure (34, 45, 46, 49, 80, 81). This treatment strategy allows for targeting of the intact metastasis, which allows for more precise SRS targeting compared to postoperative treatment. In the postoperative setting, a CTV is generated, which is dependent on resection cavity dynamics (82–84). Additionally, while preoperative SRS volumes may often be smaller than what would be expected in the postoperative setting, relaxation of the resection cavity in the interval between surgery and SRS may lead to smaller SRS volumes. A radiosurgical plan for a patient who underwent preoperative SRS is presented in Figure 2.

#### Local tumor control

As noted in the preceding sections, while postoperative SRS is associated with acceptable rates of local control, with the highest control rates for smaller resection cavities (8, 13, 16). In 2016, Patel et al. published a multicenter retrospective study comparing patients who underwent preoperative and postoperative SRS for a brain metastasis (81). The 1 year cumulative incidence of local recurrence was 15.9% vs. 12.6% in the preoperative and postoperative SRS groups, respectively (*p* = 0.33). A follow-up multicenter study that evaluated 242 patients with 253 index lesions who were treated with preoperative SRS observed 1 and 2 year local recurrence rates of 15% and 17.9%, respectively (49). Taken together, these findings suggest that preoperative SRS does not compromise local control rates when compared to postoperative SRS.

#### Radionecrosis

Radionecrosis is a potential complication following SRS that has been observed in 5%–25% of published reports (41, 85–89). The variability in reported incidence is largely due to different definitions in published studies, which incorporate pathologic and/or radiographic characteristics. Approximately 50% of radionecrosis cases are symptomatic and first-line management involves the use of corticosteroids or other systemic agents, such as bevacizumab and pentoxifylline (90). Patients who are refractory to pharmacologic managements often undergo surgical resection or laser interstitial thermal ablation (34). The volume of healthy brain irradiated during SRS is correlated with an increased risk of developing radionecrosis (8, 41, 42). A commonly used dosimetric parameter for single fraction SRS is to minimize this risk is keeping the volume of brain receiving 12 Gy or more to < 10 cm^3^ (V12 Gy < 10 cm^3^). A frequently employed mitigation strategy in the setting of larger lesions, where the brain V12 Gy would be exceeded with single fraction SRS is fractionated radiosurgery (42). In 2013, Minniti et al., published a comparative study where 289 patients with brain metastases > 2 cm in diameter were treated with single-fraction SRS or fractionated SRS (27 Gy in 3 fractions) (42). In the fractionated SRS group, the V18 Gy was found to be the most predictive parameter for radionecrosis with a risk of 5% and 14% for V18 Gy ≤ 30.2 cm^3^ and >30.2 cm^3^, respectively.

When treating postoperative cavities, it is common to incorporate a 1–2 mm CTV margin. Additionally, coverage of the surgical tract, as well as incorporation of a CTV margin along the dura and venous sinuses are recommended in certain situations (40). When incorporating a CTV into the SRS treatment volume, the amount of irradiated healthy brain significantly increases, which therefore poses an increased risk of radionecrosis. Patel et al. reported symptomatic radionecrosis rates at 1 year of 14.6% vs. 1.5% for postoperative and preoperative SRS, respectively (*p* = 0.01). Therefore, these findings suggest that rates of symptomatic radionecrosis are lower with preoperative than postoperative SRS.

#### Leptomeningeal disease

As noted in the preceding sections, LMD is frequently seen following resection and postoperative SRS; additionally, patients with breast cancer carry a higher risk (45, 46, 80). Studies have reported that the risk of developing LMD is higher following postoperative SRS than postoperative WBRT (49). It has been hypothesized that this is due to seeding of the CSF space during surgical resection, which would normally be sterilized with administration of WBRT (49, 80, 81, 91).

Patel et al. reported LMD rates at 1 year of 3.2% vs. 8.3% and at 2 years of 3.2% vs. 16.6% for pre-operative vs. post-operative SRS, respectively (*p* = 0.01) (81). Preoperative SRS may provide field sterilization to reduce the risk of tumor seeding and the subsequent development of LMD, which may occur when postoperative SRS is given postoperatively. Patel et al. conducted an additional study that compared preoperative SRS to postoperative WBRT (92). They observed LMD rates of 3.5% vs. 9.0% for the preoperative SRS and WBRT groups, respectively (*p* = 0.66). Prabhu et al. noted that the median OS following LMD diagnosis was 6.9 months vs. 1.2 months in patients with nLMD and cLMD, respectively (*p* = 0.05) (49). Additionally, the median OS for patients diagnosed with LMD who received salvage treatment was 11.3 months vs. 2.8 months in patients with nLMD and cLMD, respectively (*p* = 0.38). These findings appear to suggest the following: (1) the risk of LMD is significantly lower with preoperative SRS compared to postoperative SRS; (2) rates of LMD with preoperative SRS are not significantly different than those with postoperative WBRT; (3) nLMD is a unique failure pattern observed with postoperative SRS that is associated with better outcomes when compared to cLMD.

#### Other considerations

Preclinical studies have suggested that ionizing radiation (RT) has the ability to enhance anti-tumor immunity by acting as an in-situ vaccine (93). Additionally, studies have shown that ablative doses of RT (e.g., SRS) lead to increased antigen presentation and CD8^+^ T cell activation (5, 6, 93–97). In recent years, combining SRS with immune checkpoint inhibitors (ICI) has been a widely studied. Multiple reports have suggested that SRS and ICI are able to synergize to further enhance anti-tumor immunity, particularly when these therapies are administered within 4-weeks of one another (5–7, 65, 98–101). Preoperative SRS would permit tissue analysis following RT, which can allow for characterization of DNA repair pathways and other changes in the tumor microenvironment in response to SRS. These tissue analyses can aid in our understanding of the role that SRS plays in anti-tumor immunity, as well as identify novel therapeutic targets. Resection cavity dynamics following surgery is an important consideration in the postoperative setting (82, 102–104). This has been shown to be particularly evident in larger irregularly shaped cavities and lesions that were associated with significant amounts of edema preoperatively. Intraoperative radiotherapy (IORT) in the setting of resected metastases and glioblastoma is a novel technique that continues to expand and is associated with less cavity shrinkage compared to SRS following treatment (82–84). Both preoperative SRS and IORT allow for elimination of time to initiation of radiation, minimization of target uncertainty in a resection cavity, and dose escalation (105).

#### Disadvantages

While preoperative SRS has many advantages, it has disadvantages as well. First, preoperative SRS does not permit for pathologic tissue diagnosis prior to administering treatment. However, patients with brain metastases frequently have pathologic disease confirmation from biopsy of the primary tumor or an extracranial metastatic site prior to SRS. Second, in patients who have significant mass effect and symptoms from a brain metastasis, preoperative SRS is likely not appropriate, as the delay between preoperative SRS and surgical resection could cause an unacceptable risk to the patient. Therefore, these patients should be treated with surgical resection expeditiously.

#### Ongoing and planned clinical trials

There are multiple phase 3 clinical trials that are underway or planned that are comparing preoperative to postoperative SRS for brain metastases. Trials are underway at both the Mayo Clinic (NCT03750227) and the MD Anderson Cancer Center (NCT03741673). Additionally, NRG Oncology has recently opened the BN012 trial (NCT05438212) (106), which is a phase 3 randomized cooperative group trial comparing preoperative to postoperative SRS.

## High grade glioma and glioblastoma

High grade glioma and glioblastoma are primary brain tumors that arise from astrocytes, ependymal cells, and oligodendrocytes. Glioblastoma the most common primary brain tumor, accounting for approximately 50% of all primary brain tumor diagnoses in the United States (107, 108). Glioblastoma is highly resistant to treatment and is associated with a dismal prognosis (109, 110). Despite maximal optimal treatment, the median OS ranges from 15 to 21 months with a 5 year OS of <5% (111–115) for favorable patients who are able to undergo resection. Thus, given the poor prognosis associated with glioblastoma, novel treatments to improve the therapeutic ratio are sorely needed.

### Postoperative radiosurgery

Studies have demonstrated that most glioblastoma recurrences occur within 2 cm of the resection cavity, thus there has been interest in radiation dose escalation (59, 60, 116). Radiation Therapy Oncology Group (RTOG) 9305 was a randomized trial of 203 patients with supratentorial glioblastoma who received 60 Gy in 30 fractions and BCNU with or without the addition of an SRS boost (58). The SRS dose was based on the maximum tumor diameter, as recommended in RTOG 9005 (117). With a median follow-up of 61 months, the median OS was 13.5 months vs. 13.6 months, in the SRS and no SRS groups, respectively. Thus, postoperative SRS is not recommended in the setting of glioblastoma.

### Preoperative radiosurgery

Due to patterns of failure on glioblastoma, there has long been an interest in dose intensification; however, studies have yielded disappointing results (11, 110). BN001 is a randomized controlled trial that is comparing standard of care chemoradiotherapy to dose-escalated RT in the management of glioblastoma. Gondi et al. presented preliminary results at the 2020 Annual Meeting of the American Society of Radiation Oncology (118). No meaningful improvements in OS or other patient outcomes were observed. Preoperative SRS is therefore an attractive and novel approach to deliver intensified doses of RT in these patients.

**Figure 3:**
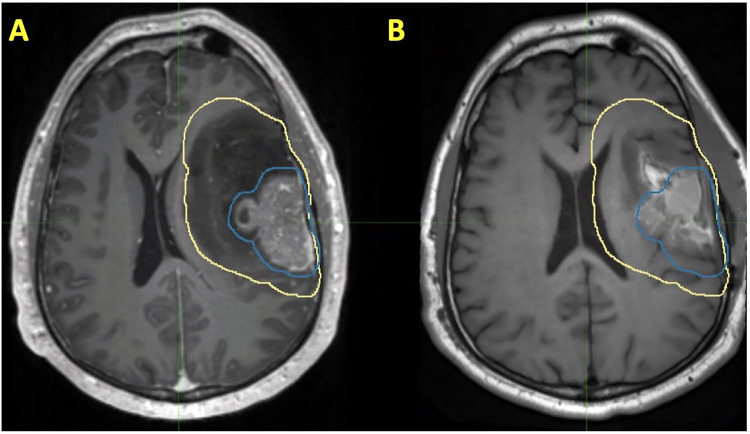
Patient with a history of glioblastoma, where the volume encircled in blue represents a preoperative SRS volume and the volume encircled in yellow represents a postoperative radiotherapy volume. Each of these volumes are shown on (**A**) preoperative T1 post contrast MRI and (**B**) T1 postoperative T1 post contrast MRI.

While there is a paucity of clinical data involving the use of preoperative SRS in the management of glioblastoma, much of the theoretical advantages can be extrapolated from what has been observed in the preclinical setting and what has been observed with brain metastases. First, intact glioblastoma may have higher rates of oxygenation when compared to postoperative tissues, which may result in more double-stranded DNA breaks (109). However, this hypothesis needs to be further validated. Second, post-SRS tissue analysis may permit characterization of cellular pathways in response to RT and can aid in the development of novel therapeutic agents. The risk of LMD in the setting of glioblastoma is approximately 4% and carries a grim prognosis (119, 120). Therefore, while preoperative SRS would be expected to decrease the risk of LMD, the extent at which it does so may be minimal. Preoperative SRS and postoperative RT treatment volumes in a patient with glioblastoma are presented in Figure 3.

#### Immunogenic effects of preoperative SRS

One of the major potential advantages of utilizing preoperative SRS in glioblastoma and high grade glioma is the ability to enhance anti-tumor immunity. A study by Klein et al., which exposed glioblastoma specimens to increasing doses of RT observed an increase in the expression of major histocompatibility class I antigen expression in response to treatment (62). This suggests that RT may cause enhanced CD8^+^ T cell responses against the tumor. A study by Newcomb et al. assessed the impact of WBRT and vaccination on a murine GL261 glioma model (61). While each of these treatments did not demonstrate a significant impact on OS when administered alone, combining WBRT and vaccination results in a long-term OS increase of 40%–80%. However, GL261 glioma models are suboptimal for studying immunotherapy in gliomas. A study by Zeng et al. in 2012 evaluated the impact of SRS and anti-PD-1 therapy on a murine glioblastoma model (121). While the OS rates were approximately 25 days in each the control, SRS, and anti-PD-1 groups, an OS of 53 days was observed in the combination therapy group. These findings suggest that SRS and anti-PD-1 therapy may synergistically enhance anti-tumor immunity in glioblastoma.

The timing of surgery following preoperative SRS is also an important consideration from an immunologic standpoint. A study by De La Maza et al. that utilized a murine mesothelioma model demonstrated that performing surgery 7 days after completing RT resulted in lower tumor regrowth rates and enhanced tumor rejection at 90 days following treatment completion (122). These findings were not observed when surgery occurred 1-day following completion of RT. Additionally, these findings were felt to be immunologic in nature, as mice depleted of CD4^+^ T cells had a markedly diminished response. Surgery alone has demonstrated an immunogenic effect on glioblastoma, Khalsa et al. showed that surgery might improve antitumor responses by increasing the presence of activated microglia, SiglecF + macrophages, T cells, while decreasing resident macrophages (123).

### Ongoing trials

The NeoGlioma study (NCT05030298) is a prospective clinical trial at the Mayo Clinic that will be investigating the role of preoperative SRS in high grade glioma. Patients will undergo surgical resection within 14 days of SRS (124). Patients in the preoperative SRS arm will undergo stereotactic biopsy prior to radiosurgery. The gross tumor volume (GTV) will be defined as residual contrast-enhancing tumor on thin slice T1-postcontrast MRI; edema will not be included. A 3 mm volumetric expansion with then be generated on the GTV to create a planning tumor volume (PTV). A clinical target volume will not be utilized and an SRS dose of 10 Gy will be prescribed to the PTV. Steroid use is at the discretion of the treating physician. The risks and benefits of steroid administration should be carefully weighed against one another, as they can provide symptom relief but also are immunosuppressive (125–127).

## Conclusion

Preoperative SRS is a treatment paradigm that has multiple significant advantages when compared to postoperative SRS in the management of brain metastases. Multiple retrospective reports have demonstrated excellent rates of local control, as well as lower rates of radionecrosis and LMD. Ongoing and planned phase 3 trials may further validate these findings. Preclinical data has suggested that preoperative SRS in the setting of high grade glioma and glioblastoma may enhance anti-tumor immune responses, which can potentially lead to improved patient outcomes. We eagerly await the results of the NeoGlioma study to better evaluate this hypothesis.

## References

[1] Leksell L. The stereotaxic method and radiosurgery of the brain. Acta Chir Scand. (1951) 102(4):316–9. Epub 1951/12/13. 14914373

[2] Leksell L. Stereotactic radiosurgery. J Neurol Neurosurg Psychiatry. (1983) 46(9):797–803. 10.1136/jnnp.46.9.797 6352865 PMC1027560

[3] Singh R Lehrer EJ Ko S Peterson J Lou Y Porter AB Brain metastases from non-small cell lung cancer with egfr or alk mutations: a systematic review and meta-analysis of multidisciplinary approaches. Radiother Oncol. (2020) 144:165–79. 10.1016/j.radonc.2019.11.010 31812932

[4] Dutta SW Kowalchuk RO Trifiletti DM Peach MS Sheehan JP Larner JM Stereotactic shifts during frame-based image-guided stereotactic radiosurgery: clinical measurements. Int J Radiat Oncol Biol Phys. (2018) 102(4):895–902. 10.1016/j.ijrobp.2018.05.042 30170871

[5] Lehrer EJ McGee HM Peterson JL Vallow L Ruiz-Garcia H Zaorsky NG Stereotactic radiosurgery and immune checkpoint inhibitors in the management of brain metastases. Int J Mol Sci. (2018) 19(10). 10.3390/ijms19103054 30301252 PMC6213912

[6] Lehrer EJ McGee HM Sheehan JP Trifiletti DM. Integration of immuno-oncology with stereotactic radiosurgery in the management of brain metastases. J Neurooncol. (2021) 151(1):75–84. 10.1007/s11060-020-03427-6 32052355

[7] Lehrer EJ Peterson J Brown PD Sheehan JP Quinones-Hinojosa A Zaorsky NG Treatment of brain metastases with stereotactic radiosurgery and immune checkpoint inhibitors: an international meta-analysis of individual patient data. Radiother Oncol. (2019) 130:104–12. 10.1016/j.radonc.2018.08.025 30241791

[8] Lehrer EJ Peterson JL Zaorsky NG Brown PD Sahgal A Chiang VL Single versus multifraction stereotactic radiosurgery for large brain metastases: an international meta-analysis of 24 trials. Int J Radiat Oncol Biol Phys. (2019) 103(3):618–30. 10.1016/j.ijrobp.2018.10.038 30395902

[9] Lehrer EJ Prabhu AV Sindhu KK Lazarev S Ruiz-Garcia H Peterson JL Proton and heavy particle intracranial radiosurgery. Biomedicines. (2021) 9(1). 10.3390/biomedicines9010031 33401613 PMC7823941

[10] Lehrer EJ Snyder MH Desai BD Li CE Narayan A Trifiletti DM Clinical and radiographic adverse events after gamma knife radiosurgery for brainstem lesions: a dosimetric analysis. Radiother Oncol. (2020) 147:200–9. 10.1016/j.radonc.2020.05.010 32413528

[11] Lehrer EJ Ruiz-Garcia H Nehlsen AD Sindhu KK Estrada RS Borst GR Preoperative stereotactic radiosurgery for glioblastoma. Biology (Basel). (2022) 11(2). 10.3390/biology11020194 35205059 PMC8869151

[12] Singh R Didwania P Lehrer EJ Palmer JD Trifiletti DM Sheehan JP. Repeat stereotactic radiosurgery for locally recurrent brain metastases previously treated with stereotactic radiosurgery: a systematic review and meta-analysis of efficacy and safety. J Radiosurg SBRT. (2022) 8(1):1–10. Epub 2022/04/08. 35387405 PMC8930057

[13] Brown PD Ballman KV Cerhan JH Anderson SK Carrero XW Whitton AC Postoperative stereotactic radiosurgery compared with whole brain radiotherapy for resected metastatic brain disease (ncctg N107c/cec.3): a multicentre, randomised, controlled, phase 3 trial. Lancet Oncol. (2017) 18(8):1049–60. 10.1016/S1470-2045(17)30441-2 28687377 PMC5568757

[14] Brown PD Jaeckle K Ballman KV Farace E Cerhan JH Anderson SK Effect of radiosurgery alone vs radiosurgery with whole brain radiation therapy on cognitive function in patients with 1 to 3 brain metastases: a randomized clinical trial. JAMA. (2016) 316(4):401–9. 10.1001/jama.2016.9839 27458945 PMC5313044

[15] Chang EL Wefel JS Hess KR Allen PK Lang FF Kornguth DG Neurocognition in patients with brain metastases treated with radiosurgery or radiosurgery plus whole-brain irradiation: a randomised controlled trial. Lancet Oncol. (2009) 10(11):1037–44. 10.1016/S1470-2045(09)70263-3 19801201

[16] Mahajan A Ahmed S McAleer MF Weinberg JS Li J Brown P Post-Operative stereotactic radiosurgery versus observation for completely resected brain metastases: a single-centre, randomised, controlled, phase 3 trial. Lancet Oncol. (2017) 18(8):1040–8. 10.1016/S1470-2045(17)30414-X 28687375 PMC5560102

[17] Trifiletti DM Dutta SW Lee CC Sheehan JP. Pituitary tumor radiosurgery. Prog Neurol Surg. (2019) 34:149–58. 10.1159/000493059 31096230

[18] Trifiletti DM Lee CC Kano H Cohen J Janopaul-Naylor J Alonso-Basanta M Stereotactic radiosurgery for brainstem metastases: an international cooperative study to define response and toxicity. Int J Radiat Oncol Biol Phys. (2016) 96(2):280–8. 10.1016/j.ijrobp.2016.06.009 27478166 PMC5014646

[19] Flickinger JC Kondziolka D Maitz AH Lunsford LD. Analysis of neurological sequelae from radiosurgery of arteriovenous malformations: how location affects outcome. Int J Radiat Oncol Biol Phys. (1998) 40(2):273–8. 10.1016/s0360-3016(97)00718-9 9457809

[20] Flickinger JC Kondziolka D Maitz AH Lunsford LD. Gamma knife radiosurgery of imaging-diagnosed intracranial meningioma. Int J Radiat Oncol Biol Phys. (2003) 56(3):801–6. 10.1016/s0360-3016(03)00126-3 12788188

[21] Flickinger JC Kondziolka D Niranjan A Lunsford LD. Results of acoustic neuroma radiosurgery: an analysis of 5 Years’ experience using current methods. J Neurosurg. (2001) 94(1):1–6. 10.3171/jns.2001.94.1.0001 11147876

[22] Flickinger JC Pollock BE Kondziolka D Lunsford LD. A dose-response analysis of arteriovenous malformation obliteration after radiosurgery. Int J Radiat Oncol Biol Phys. (1996) 36(4):873–9. 10.1016/s0360-3016(96)00316-1 8960516

[23] Kondziolka D Kano H Kanaan H Madhok R Mathieu D Flickinger JC Stereotactic radiosurgery for radiation-induced meningiomas. Neurosurgery. (2009) 64(3):463–9. discussion 9-70. 10.1227/01.NEU.0000336765.85922.D9 19240608

[24] Kondziolka D Madhok R Lunsford LD Mathieu D Martin JJ Niranjan A Stereotactic radiosurgery for convexity meningiomas. J Neurosurg. (2009) 111(3):458–63. 10.3171/2008.8.JNS17650 19199473

[25] Kondziolka D Nathoo N Flickinger JC Niranjan A Maitz AH Lunsford LD. Long-Term results after radiosurgery for benign intracranial tumors. Neurosurgery. (2003) 53(4):815–21. discussion 21-2. 10.1093/neurosurgery/53.4.815 14519213

[26] Kondziolka D Patel AD Kano H Flickinger JC Lunsford LD. Long-Term outcomes after gamma knife radiosurgery for meningiomas. Am J Clin Oncol. (2016) 39(5):453–7. 10.1097/COC.0000000000000080 24755664

[27] Kowalchuk RO Niranjan A Lee C Yang H Liscak R Guesynova K Reirradiation with stereotactic radiosurgery after local or marginal recurrence of brain metastases from previous radiosurgery. Int J Radiat Oncol Biol Phys. (2021) 112:726–34. 10.1016/j.ijrobp.2021.10.008 34644606

[28] Kowalchuk RO Shepard MJ Sheehan K Sheehan D Faramand A Niranjan A Treatment of who grade 2 meningiomas with stereotactic radiosurgery: identification of an optimal group for srs using rpa. Int J Radiat Oncol Biol Phys. (2021) 110(3):804–14. 10.1016/j.ijrobp.2021.01.048 33548341

[29] Sheehan JP Pouratian N Steiner L Laws ER Vance ML. Gamma knife surgery for pituitary adenomas: factors related to radiological and endocrine outcomes. J Neurosurg. (2011) 114(2):303–9. 10.3171/2010.5.JNS091635 20540596

[30] Sheehan JP Starke RM Kano H Barnett GH Mathieu D Chiang V Gamma knife radiosurgery for posterior Fossa meningiomas: a multicenter study. J Neurosurg. (2015) 122(6):1479–89. 10.3171/2014.10.JNS14139 25859812

[31] Sheehan JP Starke RM Kano H Kaufmann AM Mathieu D Zeiler FA Gamma knife radiosurgery for sellar and parasellar meningiomas: a multicenter study. J Neurosurg. (2014) 120(6):1268–77. 10.3171/2014.2.JNS13139 24678777

[32] Sheehan JP Starke RM Mathieu D Young B Sneed PK Chiang VL Gamma knife radiosurgery for the management of nonfunctioning pituitary adenomas: a multicenter study. J Neurosurg. (2013) 119(2):446–56. 10.3171/2013.3.JNS12766 23621595

[33] Sheehan JP Williams BJ Yen CP. Stereotactic radiosurgery for who grade I meningiomas. J Neurooncol. (2010) 99(3):407–16. 10.1007/s11060-010-0363-x 20734218

[34] Routman DM Yan E Vora S Peterson J Mahajan A Chaichana KL Preoperative stereotactic radiosurgery for brain metastases. Front Neurol. (2018) 9:959. 10.3389/fneur.2018.00959 30542316 PMC6277885

[35] Trifiletti DM Ruiz-Garcia H Quinones-Hinojosa A Ramakrishna R Sheehan JP. The evolution of stereotactic radiosurgery in neurosurgical practice. J Neurooncol. (2021) 151(3):451–9. 10.1007/s11060-020-03392-0 33611711

[36] Park HS Wang EH Rutter CE Corso CD Chiang VL Yu JB. Changing practice patterns of gamma knife versus linear accelerator-based stereotactic radiosurgery for brain metastases in the us. J Neurosurg. (2016) 124(4):1018–24. 10.3171/2015.4.JNS1573 26473783

[37] Patchell RA Tibbs PA Walsh JW Dempsey RJ Maruyama Y Kryscio RJ A randomized trial of surgery in the treatment of single metastases to the brain. N Engl J Med. (1990) 322(8):494–500. 10.1056/NEJM199002223220802 2405271

[38] Patchell RA Tibbs PA Regine WF Dempsey RJ Mohiuddin M Kryscio RJ Postoperative radiotherapy in the treatment of single metastases to the brain: a randomized trial. JAMA. (1998) 280(17):1485–9. 10.1001/jama.280.17.1485 9809728

[39] Lehrer EJ Jones BM Dickstein DR Green S Germano IM Palmer JD The cognitive effects of radiotherapy for brain metastases. Front Oncol. (2022) 12:893264. 10.3389/fonc.2022.893264 35847842 PMC9279690

[40] Soliman H Ruschin M Angelov L Brown PD Chiang VLS Kirkpatrick JP Consensus contouring guidelines for postoperative completely resected cavity stereotactic radiosurgery for brain metastases. Int J Radiat Oncol Biol Phys. (2018) 100(2):436–42. 10.1016/j.ijrobp.2017.09.047 29157748

[41] Minniti G Clarke E Lanzetta G Osti MF Trasimeni G Bozzao A Stereotactic radiosurgery for brain metastases: analysis of outcome and risk of brain radionecrosis. Radiat Oncol. (2011) 6:48. 10.1186/1748-717X-6-48 21575163 PMC3108308

[42] Minniti G Esposito V Clarke E Scaringi C Lanzetta G Salvati M Multidose stereotactic radiosurgery (9 gy X 3) of the postoperative resection cavity for treatment of large brain metastases. Int J Radiat Oncol Biol Phys. (2013) 86(4):623–9. 10.1016/j.ijrobp.2013.03.037 23683828

[43] Minniti G Scaringi C Paolini S Lanzetta G Romano A Cicone F Single-Fraction versus multifraction (3 X 9 gy) stereotactic radiosurgery for large (>2 cm) brain metastases: a comparative analysis of local control and risk of radiation-induced brain necrosis. Int J Radiat Oncol Biol Phys. (2016) 95(4):1142–8. 10.1016/j.ijrobp.2016.03.013 27209508

[44] Lehrer EJ Gurewitz J Bernstein K Patel D Kondziolka D Niranjan A Radiation necrosis in renal cell carcinoma brain metastases treated with checkpoint inhibitors and radiosurgery: an international multicenter study. Cancer. (2022) 128:1429–38. 10.1002/cncr.34087 35077586

[45] Turner BE Prabhu RS Burri SH Brown PD Pollom EL Milano MT Nodular leptomeningeal disease-a distinct pattern of recurrence after postresection stereotactic radiosurgery for brain metastases: a multi-institutional study of interobserver reliability. Int J Radiat Oncol Biol Phys. (2020) 106(3):579–86. 10.1016/j.ijrobp.2019.10.002 31605786 PMC9527087

[46] Atalar B Modlin LA Choi CY Adler JR Gibbs IC Chang SD Risk of leptomeningeal disease in patients treated with stereotactic radiosurgery targeting the postoperative resection cavity for brain metastases. Int J Radiat Oncol Biol Phys. (2013) 87(4):713–8. 10.1016/j.ijrobp.2013.07.034 24054875

[47] Foreman PM Jackson BE Singh KP Romeo AK Guthrie BL Fisher WS Postoperative radiosurgery for the treatment of metastatic brain tumor: evaluation of local failure and leptomeningeal disease. J Clin Neurosci. (2018) 49:48–55. 10.1016/j.jocn.2017.12.009 29248376 PMC5805568

[48] Marcrom SR Foreman PM Colvin TB McDonald AM Kirkland RS Popple RA Focal management of large brain metastases and risk of leptomeningeal disease. Adv Radiat Oncol. (2020) 5(1):34–42. 10.1016/j.adro.2019.07.016 32051888 PMC7004932

[49] Prabhu RS Dhakal R Vaslow ZK Dan T Mishra MV Murphy ES Preoperative radiosurgery for resected brain metastases: the props-bm multicenter cohort study. Int J Radiat Oncol Biol Phys. (2021) 111(3):764–72. 10.1016/j.ijrobp.2021.05.124 34058254

[50] Brennan C Yang TJ Hilden P Zhang Z Chan K Yamada Y A phase 2 trial of stereotactic radiosurgery boost after surgical resection for brain metastases. Int J Radiat Oncol Biol Phys. (2014) 88(1):130–6. 10.1016/j.ijrobp.2013.09.051 24331659 PMC5736310

[51] Bander ED Yuan M Reiner AS Panageas KS Ballangrud AM Brennan CW Durable 5-year local control for resected brain metastases with early adjuvant srs: the effect of timing on intended-field control. Neurooncol Pract. (2021) 8(3):278–89. 10.1093/nop/npab005 34055375 PMC8153823

[52] Roth O'Brien DA Kaye SM Poppas PJ Mahase SS An A Christos PJ Time to administration of stereotactic radiosurgery to the cavity after surgery for brain metastases: a real-world analysis. J Neurosurg. (2021) 135:1–11. 10.3171/2020.10.JNS201934 34049277

[53] Roth O'Brien DA Poppas P Kaye SM Mahase SS An A Christos PJ Timing of adjuvant fractionated stereotactic radiosurgery affects local control of resected brain metastases. Pract Radiat Oncol. (2021) 11(3):e267–e75. 10.1016/j.prro.2021.01.011 33578001

[54] van Hagen P Hulshof MC van Lanschot JJ Steyerberg EW van Berge Henegouwen MI Wijnhoven BP Preoperative chemoradiotherapy for esophageal or junctional cancer. N Engl J Med. (2012) 366(22):2074–84. 10.1056/NEJMoa1112088 22646630

[55] Sauer R Becker H Hohenberger W Rodel C Wittekind C Fietkau R Preoperative versus postoperative chemoradiotherapy for rectal cancer. N Engl J Med. (2004) 351(17):1731–40. 10.1056/NEJMoa040694 15496622

[56] Bahadoer RR Dijkstra EA van Etten B Marijnen CAM Putter H Kranenbarg EM Short-Course radiotherapy followed by chemotherapy before total mesorectal excision (tme) versus preoperative chemoradiotherapy, tme, and optional adjuvant chemotherapy in locally advanced rectal cancer (rapido): a randomised, open-label, phase 3 trial. Lancet Oncol. (2021) 22(1):29–42. 10.1016/S1470-2045(20)30555-6 33301740

[57] Garcia-Aguilar J Patil S Gollub MJ Kim JK Yuval JB Thompson HM Organ preservation in patients with rectal adenocarcinoma treated with total neoadjuvant therapy. J Clin Oncol. (2022) 40:JCO2200032. 10.1200/JCO.22.00032 PMC9362876 35483010

[58] Souhami L Seiferheld W Brachman D Podgorsak EB Werner-Wasik M Lustig R Randomized comparison of stereotactic radiosurgery followed by conventional radiotherapy with carmustine to conventional radiotherapy with carmustine for patients with glioblastoma Multiforme: report of radiation therapy oncology group 93-05 protocol. Int J Radiat Oncol Biol Phys. (2004) 60(3):853–60. 10.1016/j.ijrobp.2004.04.011 15465203

[59] Redmond KJ Mehta M. Stereotactic radiosurgery for glioblastoma. Cureus. (2015) 7(12):e413. 10.7759/cureus.413 26848407 PMC4725736

[60] Tsao MN Mehta MP Whelan TJ Morris DE Hayman JA Flickinger JC The American society for therapeutic radiology and oncology (astro) evidence-based review of the role of radiosurgery for malignant glioma. Int J Radiat Oncol Biol Phys. (2005) 63(1):47–55. 10.1016/j.ijrobp.2005.05.024 16111571

[61] Newcomb EW Demaria S Lukyanov Y Shao Y Schnee T Kawashima N The combination of ionizing radiation and peripheral vaccination produces long-term survival of mice bearing established invasive Gl261 gliomas. Clin Cancer Res. (2006) 12(15):4730–7. 10.1158/1078-0432.CCR-06-0593 16899624

[62] Klein B Loven D Lurie H Rakowsky E Nyska A Levin I The effect of irradiation on expression of hla class I antigens in human brain tumors in culture. J Neurosurg. (1994) 80(6):1074–7. 10.3171/jns.1994.80.6.1074 8189262

[63] Barnholtz-Sloan JS Sloan AE Davis FG Vigneau FD Lai P Sawaya RE. Incidence proportions of brain metastases in patients diagnosed (1973 to 2001) in the metropolitan detroit cancer surveillance system. J Clin Oncol. (2004) 22(14):2865–72. 10.1200/JCO.2004.12.149 15254054

[64] Kohler BA Ward E McCarthy BJ Schymura MJ Ries LA Eheman C Annual report to the nation on the Status of cancer, 1975-2007, featuring tumors of the brain and other nervous system. J Natl Cancer Inst. (2011) 103(9):714–36. 10.1093/jnci/djr077 21454908 PMC3086878

[65] Lehrer EJ Stoltzfus KC Jones BM Gusani NJ Walter V Wang M Trends in diagnosis and treatment of metastatic cancer in the United States. Am J Clin Oncol. (2021) 44:572–9. 10.1097/COC.0000000000000866 34560720

[66] Singh R Stoltzfus KC Chen H Louie AV Lehrer EJ Horn SR Epidemiology of synchronous brain metastases. Neurooncol Adv. (2020) 2(1):vdaa041. 10.1093/noajnl/vdaa041 32363344 PMC7182307

[67] Nabors LB Portnow J Ammirati M Baehring J Brem H Butowski N Nccn practice guidelines in oncology - central nervous system cancers, version 1.2017. J National Compregensive Cancer Network. (2017) 15:1331–45. 10.6004/jnccn.2017.0166 29118226

[68] Arvold ND Lee EQ Mehta MP Margolin K Alexander BM Lin NU Updates in the management of brain metastases. Neuro Oncol. (2016) 18(8):1043–65. 10.1093/neuonc/now127 27382120 PMC4933491

[69] Nieder C Spanne O Mehta MP Grosu AL Geinitz H. Presentation, patterns of care, and survival in patients with brain metastases: what has changed in the last 20 years? Cancer. (2011) 117(11):2505–12. 10.1002/cncr.25707 24048799

[70] Tawbi HA Forsyth PA Algazi A Hamid O Hodi FS Moschos SJ Combined nivolumab and ipilimumab in melanoma metastatic to the brain. N Engl J Med. (2018) 379(8):722–30. 10.1056/NEJMoa1805453 30134131 PMC8011001

[71] Reck M Rodriguez-Abreu D Robinson AG Hui R Csoszi T Fulop A Pembrolizumab versus chemotherapy for pd-L1-positive non-small-cell lung cancer. N Engl J Med. (2016) 375(19):1823–33. 10.1056/NEJMoa1606774 27718847

[72] Gandhi L Rodriguez-Abreu D Gadgeel S Esteban E Felip E De Angelis F Pembrolizumab plus chemotherapy in metastatic non-small-cell lung cancer. N Engl J Med. (2018) 378(22):2078–92. 10.1056/NEJMoa1801005 29658856

[73] Brown PD Gondi V. Irrational fear of whole-brain radiotherapy: are we doing our patients a disservice? Cancer. (2018) 124(17):3468–73. 10.1002/cncr.31649 30192987

[74] Brown PD Gondi V Pugh S Tome WA Wefel JS Armstrong TS Hippocampal avoidance during whole-brain radiotherapy plus memantine for patients with brain metastases: phase iii trial nrg oncology Cc001. J Clin Oncol. (2020) 38(10):1019–29. 10.1200/JCO.19.02767 32058845 PMC7106984

[75] Brown PD Pugh S Laack NN Wefel JS Khuntia D Meyers C Memantine for the prevention of cognitive dysfunction in patients receiving whole-brain radiotherapy: a randomized, double-blind, placebo-controlled trial. Neuro Oncol. (2013) 15(10):1429–37. 10.1093/neuonc/not114 23956241 PMC3779047

[76] Gondi V Pugh SL Tome WA Caine C Corn B Kanner A Preservation of memory with conformal avoidance of the hippocampal neural stem-cell compartment during whole-brain radiotherapy for brain metastases (rtog 0933): a phase ii multi-institutional trial. J Clin Oncol. (2014) 32(34):3810–6. 10.1200/JCO.2014.57.2909 25349290 PMC4239303

[77] Habets EJ Dirven L Wiggenraad RG Verbeek-de Kanter A Lycklama ANGJ Zwinkels H Neurocognitive functioning and health-related quality of life in patients treated with stereotactic radiotherapy for brain metastases: a prospective study. Neuro Oncol. (2016) 18(3):435–44. 10.1093/neuonc/nov186 26385615 PMC4767242

[78] Li J Bentzen SM Li J Renschler M Mehta MP. Relationship between neurocognitive function and quality of life after whole-brain radiotherapy in patients with brain metastasis. Int J Radiat Oncol Biol Phys. (2008) 71(1):64–70. 10.1016/j.ijrobp.2007.09.059 18406884

[79] Single Fraction Stereotactic Radiosurgery Compared with Fractionated Stereotactic Radiosurgery in Treating Patients with Resected Metastatic Brain Disease. https://ClinicalTrials.gov/show/NCT04114981 Access date: June 2, 2022.

[80] Prabhu RS Turner BE Asher AL Marcrom SR Fiveash JB Foreman PM A multi-institutional analysis of presentation and outcomes for leptomeningeal disease recurrence after surgical resection and radiosurgery for brain metastases. Neuro Oncol. (2019) 21(8):1049–59. 10.1093/neuonc/noz049 30828727 PMC6682204

[81] Patel KR Burri SH Asher AL Crocker IR Fraser RW Zhang C Comparing preoperative with postoperative stereotactic radiosurgery for resectable brain metastases: a multi-institutional analysis. Neurosurgery. (2016) 79(2):279–85. 10.1227/NEU.0000000000001096 26528673

[82] Wernicke AG Lazow SP Taube S Yondorf MZ Kovanlikaya I Nori D Surgical technique and clinically relevant resection cavity dynamics following implantation of cesium-131 (cs-131) brachytherapy in patients with brain metastases. Oper Neurosurg (Hagerstown). (2016) 12(1):49–60. 10.1227/NEU.0000000000000986 27774500 PMC5068574

[83] Cifarelli CP Brehmer S Vargo JA Hack JD Kahl KH Sarria-Vargas G Intraoperative radiotherapy (iort) for surgically resected brain metastases: outcome analysis of an international cooperative study. J Neurooncol. (2019) 145(2):391–7. 10.1007/s11060-019-03309-6 31654248 PMC7007764

[84] Sarria GR Sperk E Han X Sarria GJ Wenz F Brehmer S Intraoperative radiotherapy for glioblastoma: an international pooled analysis. Radiother Oncol. (2020) 142:162–7. 10.1016/j.radonc.2019.09.023 31629553

[85] Kohutek ZA Yamada Y Chan TA Brennan CW Tabar V Gutin PH Long-Term risk of radionecrosis and imaging changes after stereotactic radiosurgery for brain metastases. J Neurooncol. (2015) 125(1):149–56. 10.1007/s11060-015-1881-3 26307446 PMC4726630

[86] Sneed PK Mendez J Vemer-van den Hoek JG Seymour ZA Ma L Molinaro AM Adverse radiation effect after stereotactic radiosurgery for brain metastases: incidence, time course, and risk factors. J Neurosurg. (2015) 123(2):373–86. 10.3171/2014.10.JNS141610 25978710

[87] Blonigen BJ Steinmetz RD Levin L Lamba MA Warnick RE Breneman JC. Irradiated volume as a predictor of brain radionecrosis after linear accelerator stereotactic radiosurgery. Int J Radiat Oncol Biol Phys. (2010) 77(4):996–1001. 10.1016/j.ijrobp.2009.06.006 19783374

[88] Schuttrumpf LH Niyazi M Nachbichler SB Manapov F Jansen N Siefert A Prognostic factors for survival and radiation necrosis after stereotactic radiosurgery alone or in combination with whole brain radiation therapy for 1-3 cerebral metastases. Radiat Oncol. (2014) 9:105. 10.1186/1748-717X-9-105 24885624 PMC4036428

[89] Kim JM Miller JA Kotecha R Xiao R Juloori A Ward MC The risk of radiation necrosis following stereotactic radiosurgery with concurrent systemic therapies. J Neurooncol. (2017) 133(2):357–68. 10.1007/s11060-017-2442-8 28434110

[90] Vellayappan BA McGranahan T Graber J Taylor L Venur V Ellenbogen R Radiation necrosis from stereotactic radiosurgery-how do we mitigate? Curr Treat Options Oncol. (2021) 22(7):57. 10.1007/s11864-021-00854-z 34097171

[91] Burri SH Ward MC Prabhu RS. Hobgoblins, iron lungs, and surgical perturbation failure? Int J Radiat Oncol Biol Phys. (2020) 108(4):996–8. 10.1016/j.ijrobp.2020.06.032 33069355

[92] Patel KR Burri SH Boselli D Symanowski JT Asher AL Sumrall A Comparing Pre-operative stereotactic radiosurgery (srs) to post-operative whole brain radiation therapy (wbrt) for resectable brain metastases: a multi-institutional analysis. J Neurooncol. (2017) 131(3):611–8. 10.1007/s11060-016-2334-3 28000105

[93] Lee Y Auh SL Wang Y Burnette B Wang Y Meng Y Therapeutic effects of ablative radiation on local tumor require Cd8+ T cells: changing strategies for cancer treatment. Blood. (2009) 114(3):589–95. 10.1182/blood-2009-02-206870 19349616 PMC2713472

[94] Dewan MZ Galloway AE Kawashima N Dewyngaert JK Babb JS Formenti SC Fractionated but not single-dose radiotherapy induces an immune-mediated abscopal effect when combined with anti-ctla-4 antibody. Clin Cancer Res. (2009) 15(17):5379–88. 10.1158/1078-0432.CCR-09-0265 19706802 PMC2746048

[95] Vanpouille-Box C Alard A Aryankalayil MJ Sarfraz Y Diamond JM Schneider RJ DNA Exonuclease Trex1 regulates radiotherapy-induced tumour immunogenicity. Nat Commun. (2017) 8:15618. 10.1038/ncomms15618 28598415 PMC5472757

[96] Vanpouille-Box C Diamond JM Pilones KA Zavadil J Babb JS Formenti SC Tgfbeta is a master regulator of radiation therapy-induced antitumor immunity. Cancer Res. (2015) 75(11):2232–42. 10.1158/0008-5472.CAN-14-3511 25858148 PMC4522159

[97] Vanpouille-Box C Pilones KA Wennerberg E Formenti SC Demaria S. In situ vaccination by radiotherapy to improve responses to anti-ctla-4 treatment. Vaccine. (2015) 33(51):7415–22. 10.1016/j.vaccine.2015.05.105 26148880 PMC4684480

[98] Chen L Douglass J Kleinberg L Ye X Marciscano AE Forde PM Concurrent immune checkpoint inhibitors and stereotactic radiosurgery for brain metastases in non-small cell lung cancer, melanoma, and renal cell carcinoma. Int J Radiat Oncol Biol Phys. (2018) 100(4):916–25. 10.1016/j.ijrobp.2017.11.041 29485071

[99] Cohen-Inbar O Shih HH Xu Z Schlesinger D Sheehan JP. The effect of timing of stereotactic radiosurgery treatment of melanoma brain metastases treated with ipilimumab. J Neurosurg. (2017) 127(5):1007–14. 10.3171/2016.9.JNS161585 28059663

[100] Kiess AP Wolchok JD Barker CA Postow MA Tabar V Huse JT Stereotactic radiosurgery for melanoma brain metastases in patients receiving ipilimumab: safety profile and efficacy of combined treatment. Int J Radiat Oncol Biol Phys. (2015) 92(2):368–75. 10.1016/j.ijrobp.2015.01.004 25754629 PMC4955924

[101] Lehrer EJ Singh R Wang M Chinchilli VM Trifiletti DM Ost P Safety and survival rates associated with ablative stereotactic radiotherapy for patients with oligometastatic cancer: a systematic review and meta-analysis. JAMA Oncol. (2021) 7(1):92–106. 10.1001/jamaoncol.2020.6146 33237270 PMC7689573

[102] Ahmed S Hamilton J Colen R Schellingerhout D Vu T Rao G Change in postsurgical cavity size within the first 30 days correlates with extent of surrounding edema: consequences for postoperative radiosurgery. J Comput Assist Tomogr. (2014) 38(3):457–60. 10.1097/RCT.0000000000000058 24681852 PMC4181559

[103] Yuan M Behrami E Pannullo S Schwartz TH Wernicke AG. The relationship between tumor volume and timing of post-resection stereotactic radiosurgery to maximize local control: a critical review. Cureus. (2019) 11(9):e5762. 10.7759/cureus.5762 31723521 PMC6825444

[104] Atalar B Choi CY Harsh G Chang SD Gibbs IC Adler JR Cavity volume dynamics after resection of brain metastases and timing of postresection cavity stereotactic radiosurgery. Neurosurgery. (2013) 72(2):180–5. discussion 5. 10.1227/NEU.0b013e31827b99f3 23149969

[105] Vargo JA Sparks KM Singh R Jacobson GM Hack JD Cifarelli CP. Feasibility of dose escalation using intraoperative radiotherapy following resection of large brain metastases compared to post-operative stereotactic radiosurgery. J Neurooncol. (2018) 140(2):413–20. 10.1007/s11060-018-2968-4 30094718 PMC6368183

[106] Oncology N Institute NC. *Comparing the Addition of Radiation Either before or after Surgery for Patients with Brain Metastases*. https://ClinicalTrials.gov/show/NCT05438212 (2022). Access date: October 2, 2022.

[107] Miller KD Ostrom QT Kruchko C Patil N Tihan T Cioffi G Brain and other central nervous system tumor statistics, 2021. CA Cancer J Clin. (2021) 71(5):381–406. 10.3322/caac.21693 34427324

[108] Ostrom QT Cioffi G Gittleman H Patil N Waite K Kruchko C Cbtrus statistical report: primary brain and other central nervous system tumors diagnosed in the United States in 2012-2016. Neuro Oncol. (2019) 21(Suppl 5):v1–v100. 10.1093/neuonc/noz150 31675094 PMC6823730

[109] Ruiz-Garcia H Ramirez-Loera C Malouff TD Seneviratne DS Palmer JD Trifiletti DM. Novel strategies for nanoparticle-based radiosensitization in glioblastoma. Int J Mol Sci. (2021) 22(18). 10.3390/ijms22189673 34575840 PMC8465220

[110] Singh R Lehrer EJ Wang M Perlow HK Zaorsky NG Trifiletti DM Dose escalated radiation therapy for glioblastoma Multiforme: an international systematic review and meta-analysis of 22 prospective trials. Int J Radiat Oncol Biol Phys. (2021) 111(2):371–84. 10.1016/j.ijrobp.2021.05.001 33991621

[111] Stupp R Hegi ME Mason WP van den Bent MJ Taphoorn MJ Janzer RC Effects of radiotherapy with concomitant and adjuvant temozolomide versus radiotherapy alone on survival in glioblastoma in a randomised phase iii study: 5-year analysis of the eortc-ncic trial. Lancet Oncol. (2009) 10(5):459–66. 10.1016/S1470-2045(09)70025-7 19269895

[112] Stupp R Mason WP van den Bent MJ Weller M Fisher B Taphoorn MJ Radiotherapy plus concomitant and adjuvant temozolomide for glioblastoma. N Engl J Med. (2005) 352(10):987–96. 10.1056/NEJMoa043330 15758009

[113] Stupp R Taillibert S Kanner A Read W Steinberg D Lhermitte B Effect of tumor-treating fields plus maintenance temozolomide vs maintenance temozolomide alone on survival in patients with glioblastoma: a randomized clinical trial. JAMA. (2017) 318(23):2306–16. 10.1001/jama.2017.18718 29260225 PMC5820703

[114] Stupp R Taillibert S Kanner AA Kesari S Steinberg DM Toms SA Maintenance therapy with tumor-treating fields plus temozolomide vs temozolomide alone for glioblastoma: a randomized clinical trial. JAMA. (2015) 314(23):2535–43. 10.1001/jama.2015.16669 26670971

[115] Cantrell JN Waddle MR Rotman M Peterson JL Ruiz-Garcia H Heckman MG Progress toward long-term survivors of glioblastoma. Mayo Clin Proc. (2019) 94(7):1278–86. 10.1016/j.mayocp.2018.11.031 31230743

[116] Wallner KE Galicich JH Krol G Arbit E Malkin MG. Patterns of failure following treatment for glioblastoma Multiforme and anaplastic astrocytoma. Int J Radiat Oncol Biol Phys. (1989) 16(6):1405–9. 10.1016/0360-3016(89)90941-3 2542195

[117] Shaw E Scott C Souhami L Dinapoli R Kline R Loeffler J Single dose radiosurgical treatment of recurrent previously irradiated primary brain tumors and brain metastases: final report of rtog protocol 90-05. Int J Radiat Oncol Biol Phys. (2000) 47(2):291–8. 10.1016/s0360-3016(99)00507-6 10802351

[118] Gondi V Pugh S Tsien C Chenevert T Gilbert M Omuro A Radiotherapy (rt) dose-intensification (di) using intensity-modulated rt (imrt) versus standard-dose (sd) rt with temozolomide (tmz) in newly diagnosed glioblastoma (gbm): preliminary results of nrg oncology Bn001. Int J Radiat Oncol Biol Phys. (2020) 108(3):S22–S3. 10.1016/j.ijrobp.2020.07.2109

[119] Mandel JJ Yust-Katz S Cachia D Wu J Liu D de Groot JF Leptomeningeal dissemination in glioblastoma; an inspection of risk factors, treatment, and outcomes at a single institution. J Neurooncol. (2014) 120(3):597–605. 10.1007/s11060-014-1592-1 25168214

[120] Akmal S Ginalis EE Patel NV Aiken R Dicpinigaitis AJ Hanft SJ. Leptomeningeal disease in glioblastoma: endgame or opportunity? J Neurooncol. (2021) 155(2):107–15. 10.1007/s11060-021-03864-x 34623599

[121] Zeng J See AP Phallen J Jackson CM Belcaid Z Ruzevick J Anti-Pd-1 blockade and stereotactic radiation produce long-term survival in mice with intracranial gliomas. Int J Radiat Oncol Biol Phys. (2013) 86(2):343–9. 10.1016/j.ijrobp.2012.12.025 23462419 PMC3963403

[122] De La Maza L Wu M Wu L Yun H Zhao Y Cattral M In situ vaccination after accelerated hypofractionated radiation and surgery in a mesothelioma mouse model. Clin Cancer Res. (2017) 23(18):5502–13. 10.1158/1078-0432.CCR-17-0438 28606922

[123] Khalsa JK Cheng N Keegan J Chaudry A Driver J Bi WL Immune phenotyping of diverse syngeneic murine brain tumors identifies immunologically distinct types. Nat Commun. (2020) 11(1):3912. 10.1038/s41467-020-17704-5 32764562 PMC7411074

[124] *Preoperative Radiosurgery for the Treatment of High Grade Glioma, the Neoglioma Study*. https://ClinicalTrials.gov/show/NCT05030298 Access date: October 2, 2022.

[125] Aldea M Orillard E Mansi L Marabelle A Scotte F Lambotte O How to manage patients with corticosteroids in oncology in the era of immunotherapy? Eur J Cancer. (2020) 141:239–51. 10.1016/j.ejca.2020.09.032 33212339

[126] Maslov DV Tawagi K Kc M Simenson V Yuan H Parent C Timing of steroid initiation and response rates to immune checkpoint inhibitors in metastatic cancer. J Immunother Cancer. (2021) 9(7). 10.1136/jitc-2020-002261 34226279 PMC8258666

[127] Petrelli F Signorelli D Ghidini M Ghidini A Pizzutilo EG Ruggieri L Association of steroids use with survival in patients treated with immune checkpoint inhibitors: a systematic review and meta-analysis. Cancers (Basel). (2020) 12(3). 10.3390/cancers12030546 PMC7139305 32120803

